# High School Math and Motivation for autistic students

**DOI:** 10.1007/s10803-022-05522-1

**Published:** 2022-04-20

**Authors:** Michael Cooper, George Farkas

**Affiliations:** School of Education, University of California Irvine, Irvine, CA United States

**Keywords:** Autism, ASD, Math, High School, Motivation, Expectancy-value

## Abstract

Analyzing data from students in the NCES High School Longitudinal Study dataset, we drew upon expectancy-value theory to examine the role of student motivation (measured by self-efficacy, identity, utility, and interest), as mediators between 9th grade math test scores and final math GPA for autistic students. In predicting final high school math GPA, math identity was the strongest predictor for autistic students with above average test scores. Findings for autistic students contrast with results for non-autistic students whose final math GPA is strongly predicted by the direct effects of 9th grade test scores. These results suggest that seeing oneself as a “math person” may be particularly influential for autistic students with higher 9th grade math performance.

**Supplementary Information:**

The online version contains supplementary material available at 10.1007/s10803-022-05522-1.

## High School Math and Motivation for Autistic Students

**Abstract**.

Analyzing data from students in the NCES High School Longitudinal Study dataset, we drew upon expectancy-value theory to examine the role of student motivation (measured by self-efficacy, identity, utility, and interest), as mediators between 9th grade math test scores and final math GPA for autistic students. In predicting final high school math GPA, math identity was the strongest predictor for autistic students with above average test scores. Findings for autistic students contrast with results for non-autistic students whose final math GPA is strongly predicted by the direct effects of 9th grade test scores. These results suggest that seeing oneself as a “math person” may be particularly influential for autistic students with higher 9th grade math performance.

## High School Math and Motivation for Autistic Students

Awareness of autism spectrum disorders has grown with its increase in prevalence, as have autism-specific interventions and educational programs (Maenner et al., 2020a, b). Many autistic students^1^ are thought to show cognitive performance similar to their nondisabled peers (Estes et al., 2011). However, although autistic students today may reach academic levels commensurate with their ability, many still appear to show academic achievement lower than their underlying cognitive ability (Estes 2011, Kim et al., 2018). This raises the issue of motivation as a potentially important factor in the ultimate educational and occupational attainment of these students.

Recent research estimates the prevalence of autism to be one in 54 (Maenner et al., 2020a, b). Under current diagnostic criteria, ASD is defined by persistent deficits in social communication and exhibition of restrictive, repetitive patterns of behaviors (Volkmar et al., 2014). ASD diagnoses in the United States include previously identified disorders such as Asperger syndrome and pervasive developmental disorder-not otherwise specified (PDD-NOS) within the spectrum. Despite this unification, it is important to recognize the heterogeneity of autistic individuals and to consider differentiating between groups of autistic individuals in analyses both to improve our understanding of autism and to more accurately capture differences within the diagnosis (Georgiades et al., 2013; Lombardo et al., 2019).

Poor academic outcomes may impede the path of autistic students to post-secondary education and subsequent occupational careers. Autistic students considered “high-functioning^2^” and their parents are shown to often have postsecondary education aspirations (Camarena & Sarigiani, 2009). However, fewer than 25% of autistic students who engage in postsecondary education or employment are found to retain those activities, meaning they fail to complete their degree or maintain employment status (Taylor et al., 2015).

Recent research suggests that aspirations to pursue STEM after high school do not significantly differ between high school students with and without disabilities (Bittinger et al., 2020). However, autistic students are more than twice as likely to be interested in STEM or to ultimately declare a STEM major when compared to students with other disabilities or to the general population (Chen & Weko 2009; Wei et al., 2012). Accordingly, autistic students, properly supported, constitute a potentially important new source of STEM workers, and STEM jobs constitute a potentially important employment basis for a successful adult life for students with this disability.

Mathematics in particular is a field highly associated with autism. There is a potential genetic link between the two, with one study showing mathematics undergraduates are seven times more likely to have autism or an immediate autistic family member when compared to students in other fields (Baron-Cohen et al., 2007). In journalism and other media, autism is heavily stereotyped with mathematical ability. Many mathematically talented historical figures are speculated to have been autistic. Popular media discussion of autism often involves mathematical savantism or splinter skills (“abilities to do a specific task that does not generalize to other tasks”) while neglecting the many other aspects and nuances of the disorder. This has helped create the perceived relationship between math and autism (Garner et al., 2015; Baron-Cohen, 2015; Radomski & Latham, 2008; Falck-Ytter & Loden, 2020). These factors led us to explore adolescent autistic students’ motivations toward mathematics success. Mathematics is a key element for STEM majors and occupations, and the connection between autism and math is a promising avenue for helping these students with disabilities to meet these future educational and career goals.

Theoretical Framework.

### Autism and Motivation

Discussion of motivation in autistic young adults has largely centered around the social motivation hypothesis of autism spectrum disorders, which argues that autistic individuals receive less innate value from typical social stimuli (Chevallier et al., 2012; Bottini, 2018). In adolescence, this may manifest in autistic students as a lack of response to typically motivating social cues such as facial expressions or body language (Bos et al., 2020). Social motivation in adolescents and adults has also been analyzed using a self-determination theory framework, a theory of motivation that proposes three intrinsic needs that determine decision-making: autonomy, competence, and relatedness (Chen et al., 2015; Deci & Ryan, 2000).

However, there have been few research studies on academic motivation for autistic young adults. Charitaki, Soulis, and Tyropoli analyzed data collected from a large (n = 200) population of primary and secondary school teachers in Greece who worked with autistic students. They found measures of self-regulation deficits to be significant and correlated with each other for autistic students (Charitaki et al., 2019). Self-regulation describes how one controls responses to new information– often involving control of emotions (Kopp, 1982). Self-regulation research explains that academics are impacted negatively for autistic students through mechanisms such as difficulty maintaining attention in class, behavioral problems, and social and emotional distractions and interpersonal difficulties in the classroom (Ashburner et al., 2010; Jahromi et al., 2013).

Georgiou et al., (2018) examined the relation between mathematics motivation and success among autistic high school students in Greece. They highlighted the importance of motivation for mathematics success in high-functioning autistic students, as well as the fact that motivation differs when compared to non-autistic peers. Dividing math motivation into components of mastery goals, performance goals, self-efficacy beliefs, fear of failure, and interest, the authors analyzed descriptive statistics of questionnaire measures of these constructs and found that autistic students have higher measures for both being interested in math and fearing failure in math when compared to a control group of their peers. The authors also called for more research in the study of “motivation in mathematics of high-functioning students with ASD” which is “in its infancy” (Georgiou et al., 2018, p104).

### Expectancy-Value Theory

Expectancy-value theory (EVT; Eccles 1983) explores achievement-related motivation that leads to achievement-related choices. The model posits that cultural milieu, socializers’ beliefs, individual aptitudes, and previous experiences influence each other and the individual’s perceptions, experiences, and memory. This complex network leads to the two primary categories that explain choices: expectations of success and subjective task value. Ability-related beliefs, similar to self-efficacy, are a part of the expectations of success construct, while utility and interest fall under the subject task value construct (Wigfield & Eccles, 2000). These final components of EVT provide a framework for empirically describing and comparing motivational differences in autistic students.

This theory has recently been employed in analyzing the determinants of outcomes for autistic students. However, these studies are largely focused on the expectations and choices made by the parents of these students (Kirby et al., 2020; Kirby 2016; Schroeder, 2016); the expectations transferred by teachers to these students (Thissen, 2012 (Master’s Thesis)); or even the motivation transferred from siblings (McHale et al., 2016). EVT has also been utilized to understand the motivation and outcomes for other groups of students, such as those with a history of reading difficulty or visual disabilities (Bergey et al., 2018; Kirk & Haegele, 2019). However, to the best of our knowledge, there have been no studies applying EVT to the motivation of autistic students themselves. It is therefore important to investigate how autistic students compare to students typically examined using the theory.

However, EVT *has* been used in researching the math-related motivation of non-disabled high school students. Guo and colleagues found self-concept (a construct that would fall into the expectancy of success construct) to have significant effects on predicting educational attainment and aspirations overall for high schoolers (Guo et al., 2015). (Lauermann et al., 2017), examining math-related career aspirations in high school, found that expectations of success and subjective task value predicted postsecondary math outcomes as far as fifteen years after graduation.

**Fig. 1:**
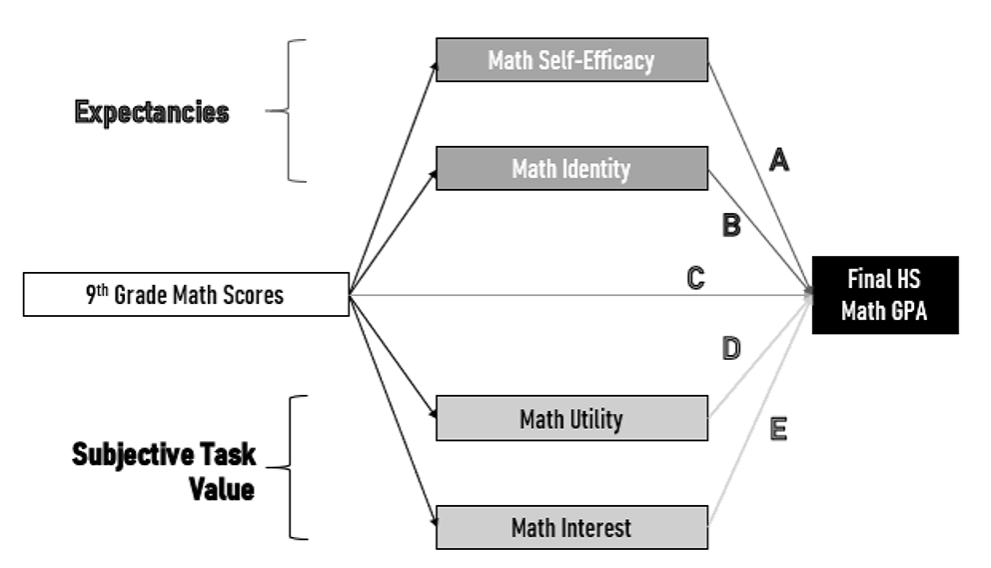
, EVT Path Diagram showing example motivational and performance inputs on final math GPA

A proposed path diagram utilizing the expectancy variables (self-efficacy and identity) and subjective task value variables (utility and interest) can be seen in Fig. 1. Ninth grade math score (a measure of initial cognitive mathematical performance) impacts final math GPA (a measure of final math performance and academic effort) both directly and through the expectancy value components of motivation.

### High-Functioning Autism and Grouping

It is quite common for research to aim to study high-functioning autistic students or students with Asperger syndrome (a diagnosis that is no longer used that was similar to what is often considered high-functioning) as a specific group of interest Alvares et al., 2020a, b; Estes et al., 2011; Georgiou et al., 2018; Jahromi et al., 2013; etc.). The heterogeneity of autism and the differences between individuals on different ends of the spectrum is important to recognize and account for when discussing autism as a whole (de Giambattista et al., 2019). However, high-functioning autism is not an official term but typically describes individuals with an intelligence quotient (IQ) of 70 or above (Lincoln et al., 1988). Yet IQ itself is an imprecise measure of functioning levels. Instead, functioning comes in various subtypes, is domain-specific, and can change over time (Alvares et al., 2019). This suggests the importance of separating groups of autistic students in a way relevant to the measures of the research – such as using test scores to group students when studying their math outcomes. Measuring descriptive statistics between such groups can also provide a better understanding of the subtypes of individuals with autism in various domains.

### Study’s Contributions

This study makes the following contributions. For the first time, we apply EVT to the motivation and decision-making of autistic high school students, in order to better understand the determinants of the mathematics achievement of these students. We do this by applying regression analysis to nationally representative data from the High School Longitudinal Study (HSLS). We also split our sample into two groups based on math test scores. We show the importance of finding subgroups within autistic students and illustrate the difference in motivation in math between those groups. We will address the following research questions:

Research Questions.

RQ1 How does the distribution of math test scores differ between autistic students and their non-autistic peers?

RQ2 What are the levels of subjective task value and expectations for success in mathematics for autistic students with below and above average test scores? How do these compare to their non-autistic peers?

RQ3. What are the most important motivational predictors, net of prior test score, of math academic achievement (final GPA) for lower and higher performing autistic and non-autistic student groups?

Methods.

### Sample

We analyze data from the NCES’s High School Longitudinal Study of 2009 (HSLS). This is a nationally representative study beginning with data from ninth graders in 944 schools in 2009 and following-up in 2012 and 2016. Data from the base year of the study includes questionnaire information from students and parents and a mathematics assessment in ninth grade (Ingels et al., 2011). Transcript data was received from the schools in the follow-up four years later in 2013 (Dalton et al., 2016).

### Grouping

Due to the varying severity of cognitive impairment and mathematical performance of autistic students, we expect a wider math score distribution when compared to non-autistic students or students with other disabilities. Some autistic students are high achievers in subjects involving math, while some others are expected to have significant difficulty, therefore we believe it is important to separate analysis into two groups of lower and higher scoring students.

**Fig. 2:**
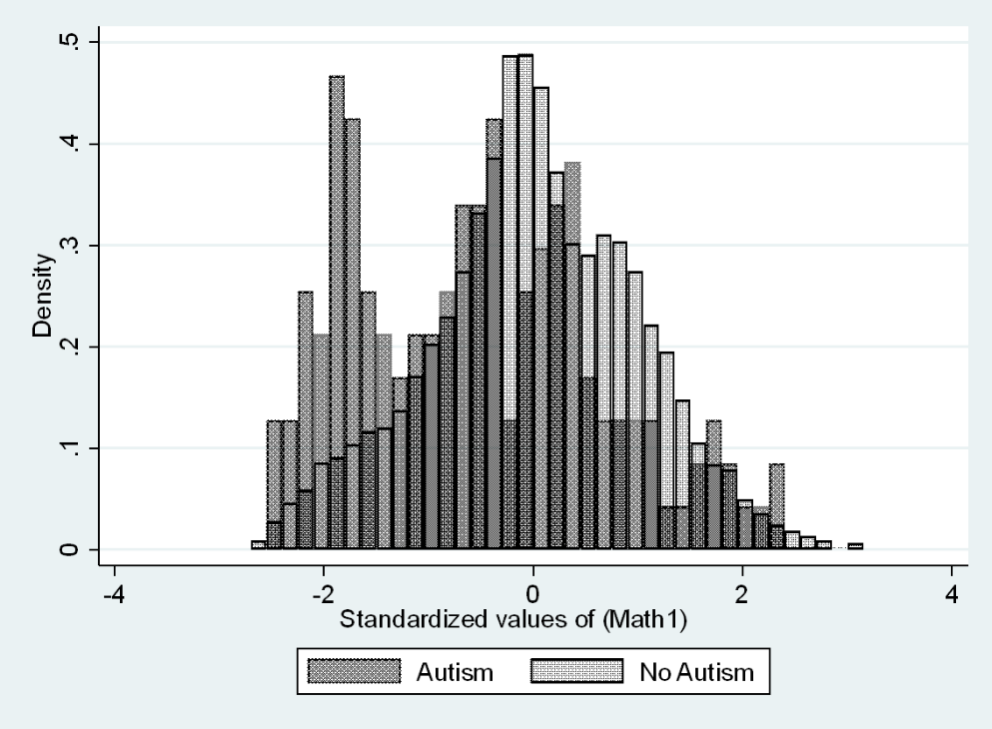
, Overlayed histograms of ninth grade math test score distributions for autistic and non-autistic students

Standardized z scores were calculated for ninth grade math test scores based on the total HSLS population. The distribution of scores for autistic and non-autistic students have been overlayed in Fig. 2. The median of math scores for autistic students corresponds with half of a standard deviation below the mean for the HSLS population as a whole, and this cutoff value was chosen to create two groups of equal size. We call these two groups “above average math scores” and “below average math scores.” The comparison non-autistic groups use the same − 0.5SD cutoff.

### Variables

*Autism.* The designation determining a student having autism in our sample comes from a parent survey taken in the base year of the study. Parents were asked if a doctor or school has ever told the parent that their child has some form of autism.

*Math Test Score.* A standardized mathematics achievement test also taken in 9th grade is used as a grouping and control variable. The assessment was a 40 min, 40 question test of algebraic reasoning and processes created for the HSLS by a mathematic advisory panel. The test was adaptive with the second stage having varying difficulty based on the student’s success in the first stage. Of the 73 different items, one was dropped due to poor statistical significance, and < 1% of respondents were dropped due to a lack of effort (fewer than six answers or “patterned marking” such as repeating the same answer for each question). Classical item analysis was used to check item performance for the questions and IRT model was used to estimate student performance. All calculations were performed by the HSLS researchers (Ingels et al., 2011). Standardized scores are utilized for this study.

*Final Math GPA.* Our outcome variable of interest was final GPA in mathematics, as reported on transcripts in the 2016 follow-up. GPA is reported on a 4.0 scale using GPA formulas also reported in all high school transcripts if not already in a 4.0 scale. Subject categories on each transcript included math. GPA remains in the 4.0 scale for this analysis (Dalton et al., 2016).

*Expectancy Value Model.* Expectancy-value theory has two constructs contributing to achievement-related decisions and performance: expectation of success and subjective task value (Eccles, 1983; Wigfield & Eccles, 2002). HSLS utilizes a very similar framework in their theoretical model for creating their survey questions simply titled “HSLS:09 base-year student survey conceptual map.” It also has two constructs contributing to academic decisions: Expectancy and Value (Ingels et al., 2011).

*Expectancy.* Expectancy is further broken up into two categories: self-efficacy and identity. For math, self-efficacy was derived from the survey questions asking students how confident or certain they are about understanding their textbook, excelling at their tests, mastering the skills, and doing well on assignments. Identity is comprised of two variables asking to what extent the student sees themselves as a “math person” and to what extent they think others see them as a “math person.” Math identity approximates attainment value or self-concept in the Eccles model.

*Subject Task Value.* Value (the term HSLS uses for subjective task value) also has two categories: utility and interest. Math utility asks students three questions about how useful they think their math class will be for everyday life, for college, and for a future career. Math interest uses 6 questions. It asks how much they are enjoying math, if they think math is a waste of time or boring, and if they are taking their math class because they enjoy math. It also includes their favorite and least favorite subjects.

All four scales were derived from exploratory factor analysis done by the HSLS team with a minimum of 0.65 for Cronbach’s alpha. The self-efficacy scale had a Cronbach’s alpha value of 0.90. The math identity scale had a Cronbach’s alpha value of 0.84. Math utility had a Cronbach’s alpha of 0.78 and math interest had a Cronbach’s alpha of 0.75. All scales are standardized for the HSLS population (Ingles et al., 2011). All surveys were taken at the beginning of 9th grade.

### Analysis Plan

All analyses were performed in STATA and separately for each of four groups of students – students with and without autism and within each group, those whose 9th grade math test scores were above and below average. To answer the first research question (RQ1) we generated histograms of math test scores for autistic students and non-autistic students and overlayed them using the same bin size.

To answer our second research question (RQ2), we calculated descriptive statistics for means and standard deviations of the expectancy-value scale items for each of our four groups. T-tests were used to compare means between groups.

To answer our third research question (RQ3), multivariate regression models were run for each of the four groups. Regression coefficients were compared between groups based on magnitude, significance, and systematic F-tests used to test for a significant difference between coefficients in two models. Systematic F-tests using interaction terms allowed us to measure if allowing the model to differ by groups for particular predictors significantly differed from constraining them. Full information maximum likelihood modeling was used to calculate the regression coefficients to account for missing data.

## Results

**Fig. 3:**
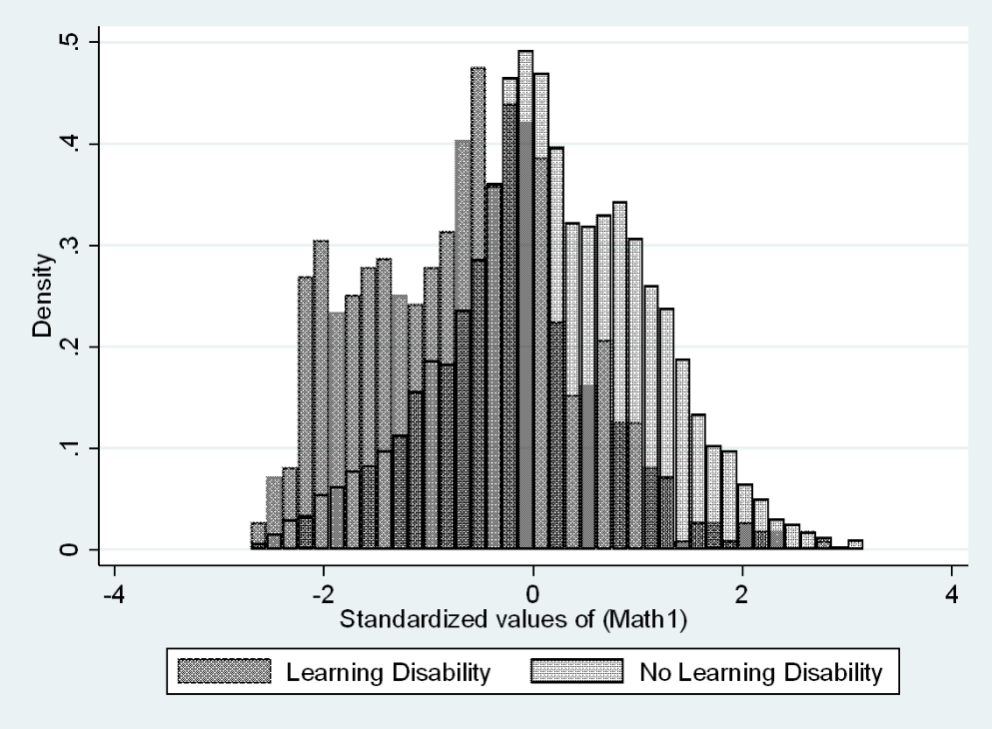
, Overlayed histograms of ninth grade math test score distributions for students with and without learning disabilities

The distribution of test scores for autistic and non-autistic 9th graders can be seen in Fig. 2. We can see that test scores for autistic students are not normally distributed and not centered around the same score as their non-autistic peers. To compare we also overlay test scores of another disability, learning disabilities, with students without disabilities in Fig. 3. The distribution of learning disability test scores is shifted left (lower test scores) and has a single peak. This is common for other disabilities tested as well, which contrasts with the multimodal distribution of autistic scores.

Predictor variables (math test scores, self-efficacy, identity, utility, and interest) are measured in the beginning of 9th grade and converted into z-scores using the entire sample. The outcome variable is final math GPA which is measured in units of GPA with a maximum of 4.0 taken from high school transcripts at the end of high school. Table 1 presents descriptive statistics for each of our groups: below average math score students with autism, below average math score students without autism, above average math score students with autism, and above average math score students without autism. Also included are the two combined groups of students with and without autism without being separated by math score. Significance for differences is always comparing the with autism and without autism groups in the table.

**Table 1:** Descriptive Statistics of Variables

			Self-Efficacy	Identity	Utility	Interest	Math Test	Final Math GPA
All Math Scores	With Autism	Mean	0.115	0.004	0.05	0.206	-0.546	2.275
	n = 157	SD	(0.980)	(1.035)	(0.953)	(0.938)	(1.180)	(0.963)
	Without Autism	Mean	0.098	0.099	-0.014	0.076	0.13	2.487
	n = 15,038	SD	(0.976)	(0.993)	(0.979)	(0.977)	(0.987)	(0.955)
		Difference	0.017	-0.095	0.064	0.13	-0.676**	-0.212**
Below Average Math Scores	With Autism	Mean	-0.175	-0.287	-0.075	0.000	-1.489	1.873
	n = 81	SD	(0.980)	(1.032)	(0.858)	(0.861)	(0.568)	(0.821)
	Without Autism	Mean	-0.297	-0.378	-0.050	-0.182	-1.153	1.810
	n = 3644	SD	(0.999)	(0.961)	(1.050)	(1.001)	(0.522)	(0.839)
		Difference	0.122	0.091	-0.025	0.182	-0.336**	0.063
Above Average Math Scores	With Autism	Mean	0.358	0.299	0.149	0.377	0.459	2.616
	n = 76	SD	(0.919)	(0.958)	(1.016)	(0.971)	(0.753)	(0.102)
	Without Autism	Mean	0.210	0.249	− 0.003	0.145	0.540	2.694
	n = 11,394	SD	(0.939)	(0.955)	(0.957)	(0.958)	(0.709)	(0.008)
		Difference	0.148	0.05	0.152	0.232*	-0.081	-0.078
Below and above average math determined from below or above median math test score of autistic students. Self-efficacy, identity, utility, interest, and math test scores all taken in the beginning of 9th grade and standardized based on the entire HSLS sample. Final math GPA remains in units of GPA and comes from transcript data at the end of high school Significance of differences from t-tests: * p < .05								
**p < .01								

Three of the four EVT variables show higher means for autistic students when compared to students without autism. This is evidence for the premise of this study, that autistic students have an unusually positive affinity for math. The largest of these differences is for math interest, but none achieve statistical significance. However, the autistic students score substantially lower than non-autistics on the math test and in final math GPA. On the math test they are .68SD lower and their math GPA is 0.21 grade points lower, which is 0.22 SD. Thus we see that the achievement test gap between autistic and non-autistic students is larger than the GPA gap.

Comparing mean values of the variables for autistic and non-autistic students with below and above average test scores, we generally see larger values by which the autistic students outscore the non-autistic students. The largest of these is math interest, where the below average scoring autistic students exceed below average students without autism by .18SD. Among students with above average test scores, the autistic students exceed the non-autistic by .23SD, and this is statistically significant. Overall, among the eight comparisons (4 EVT variables for below and above students), the autistic students have higher scores for seven of the eight. This is further evidence of the unusual affinity for math in autistic compared to non-autistic students. As for test score and GPA gaps between autistic and non-autistic students, they are smaller among students in each of the test score groupings than in the sample as a whole.

Correlations between variables are shown in Table 2 (students with autism above the diagonal and without autism below the diagonal). The highest correlations are between identity and self-efficacy, and this is the case for students with and without autism. High correlations are also seen between interest and self-efficacy. Variance inflation factors (VIF) did not present any issues with potential collinearity in using these variables to predict subsequent math GPA. Table 3 shows regressions predicting 11th grade math GPA, separately for students with and without autism, for the full population, and for those students with below and above average 9th grade math test scores. Residual plots were computed to confirm that residuals were normally distributed and independent of estimates. Note that this analysis assumes a roughly linear relationship between the estimators and GPA.

**Table 2:** Correlations of Independent Variables for Population without Autism (Bottom) and with Autism (Top)

	Math Scores	Self-Efficacy	Identity	Utility	Interest	Math GPA
Math Scores	1	0.2455	0.2841	0.1515	0.1707	0.3406
Self-Efficacy	0.3204	1	0.5847	0.4602	0.5498	0.4135
Identity	0.4093	0.5756	1	0.3837	0.5001	0.4102
Utility	0.0183	0.3524	0.3012	1	0.499	0.0514
Interest	0.2104	0.5316	0.5321	0.4401	1	0.2234
Math GPA	0.5400	0.3252	0.3590	0.0340	0.2439	1

**Table 3:** Regression Models. Effects of Motivation Predicting Final Math GPA

	Full Population			Below Average Math			Above Average Math		
	With Autism	Without Autism	Difference	With Autism	Without Autism	Difference	With Autism	Without Autism	Difference
Self-Efficacy	0.199*	0.119***	0.080*	0.210	0.072***	0.138**	0.183	0.134**	0.049
Identity	0.183*	0.084***	0.099	-0.009	0.070***	-0.079	0.361**	0.087**	0.274*
Utility	-0.193*	-0.073***	-0.120	-0.104	-0.050***	-0.054	-0.309**	-0.078**	-0.231
Interest	-0.007	0.068***	-0.075	-0.162	0.118***	-0.280*	0.170	0.061**	0.109
Test Scores	0.196**	0.447***	-0.251***	-0.251	0.256***	-0.507**	0.148	0.517**	-0.369**
Constant	2.349	2.402		1.528	2.125		2.363	2.292	
n	170	23,140		81	6056		89	17,084	
R^2^	0.262	0.33		0.098	0.089		0.238	0.258	
* p < .10 **p < .05 ***p < .01 F-tests show significant differences between coefficients									

Within the full population of autistic and non-autistic students the pattern of coefficients is similar – self-efficacy, identity, and interest are positively, whereas utility is negatively related to GPA. Test scores are also positively related to GPA, but this coefficient is much larger for non-autistic than for autistic students. This is a major finding –for autistic students the EVT expectancy variables self-efficacy and identity have positive effects comparable to those of test scores on GPA. Thus, for these students, interventions to improve these variables may be particularly effective for raising their math GPA.

For the below and above math test score groups other patterns emerge. Strikingly, for the below average group only self-efficacy has a positive relationship with GPA. Given the small sample size none of these are statistically significant. Nevertheless, the positive coefficient for self-efficacy suggests that for these students, it may be particularly important to boost their feelings of math mastery to help them improve their math grades. Comparing listwise deletion to FIML yields one coefficient change from significance in one group (math self-efficacy for the autism below average math score group from significant at the p < .05 level to no longer significant when accounting for missing data).

Among autistic students with above average test scores, the large positive coefficient for the effect of identity on GPA stands out. Self-efficacy, interest and test scores also have positive coefficients, but that for identity is approximately twice their size. These findings suggest that among autistic students with low test scores, it is most important to boost their self-efficacy – the belief that they can succeed at math. On the other hand, among autistic students with above average test scores, while self-efficacy and interest are positively related to GPA, the really important predicter is identity. For these students, who are good at math, it is particularly important that this ability has become so important that they have made it a part of their identity. Interventions to aid this process of identity formation may be important to translate math ability into a high math GPA, which will be particularly important for subsequent college enrollment and success at a STEM major and occupation.

### Discussion

For students without autism, we have found that all included elements of expectancy value theory have good predictive power for math GPA outcomes, even after controlling for prior math test scores. However, test scores remain the predominant predictor for these students. By contrast, the results are very different for autistic students. Test scores are not nearly as strong a predictor of GPA for these students. In fact, for the autistic students, whether below or above average in their math test scores, when EVT variables are controlled as predictors, test scores are not significantly related to GPA. Thus, once autistic students are separated into low and high performers, variation in math performance at the start of high school does not appear to be the main factor driving how well these students will do in math coursework by the end of high school. Instead, programs to assist these students may wish to pay particular attention to socioemotional components that impact motivation and math success to help autistic students succeed academically in math and continue to pursue STEM after high school. This adds an interesting component to our understanding of the relationship between autism and math. Math test performance does not seem to be nearly as related to outcomes in math classes.

EVT provides two general categories for understanding motivation and decision-making in education: expectancies and values. The HSLS drew upon this and included self-efficacy and identity as expectancy variables and utility and interest as value variables. For autistic students, we see higher measures of the value variables interest and utility when compared to their non-autistic peers. This difference is especially stark for math interest for students with above average test scores. These students are more interested in math and may believe it to be more useful for life and college or careers. This is in line with previous research detailing higher math interest in autistic individuals. The main previous work in this field by Georgiou and colleagues did not use an EVT framework and focused only on high-functioning students in Greece. They found similar higher interest and motivation in general for math, as have other studies (Chen & Weko, 2009; Georgious, et al., 2018; Wei et al., 2012). However, math interest fails to significantly predict final math GPA for either the below or above average math score group. Our other measure of subjective task value in this model, utility value, also fails to positively predict math test scores. In fact, having a higher utility value for math shows a negative impact on their success in math.

Expectancy measures, on the other hand, appear to be the most relevant for autistic students in math. Our analysis suggests that beliefs in their own math ability (self-efficacy) are the only positive EVT influencer on GPA. While this effect loses statistical significance after accounting for missing data, it is still the largest, and only positive effect in the model, and significantly larger than students without autism below the same math score. Intervening to improve math motivation and math success for lower performing autistic students will likely be ineffective if their self-efficacy is not addressed. These students likely need a solid belief in their foundations in math and their ability to learn things going forward – otherwise they will not succeed to the best of their abilities.

The other expectancy variable, math identity, is the strongest positive predictor of math GPA for the above average group of autistic students. While self-efficacy is still a positive predictor for this group, as is math interest, identity has a much larger and statistically significant effect on math GPA. Math identity is measured as the extent to which an individual sees themselves as a “math person” as well as how much they feel others see them as a math person. This may be in line with the idea of individuals with autism having particular interests that they are more likely to associate with themselves. The question of being a “math person” or not is more important to above average autistic students than it appears to be to their non-autistic peers – leading to changes in motivation that ultimately influence their success in their math classes. Previous work largely focuses on social motivation for autistic students. These aspects of identity – how a student sees themselves and how they perceive others see them – may show a link between social motivation and academic motivation. For autistic students with lower test scores (likely including fewer “high functioning” autistic students), their lower math performance may prevent them associating their identity with math to achieve this motivational effect – which we can see in our summary statistics for these groups.

Students with autism and above average test scores seem to respond most strongly when being a “math person” becomes part of their identity. This is the strongest positive predicter of a high math GPA for them. This group of students is mostly likely to be interested in math and capable and interested in pursuing math or STEM after high school. Understanding that how they see themselves is key to their academic success (as opposed to their 9th grade performance or how much they value math) may prove essential in helping these students succeed in school and bridge the gap to higher education and STEM careers.

Limitations.

One significant limitation of these data is the sample size of students with autism. If the predictive effects of the socioemotional variables are relatively small, this sample may be underpowered to estimate individual regression coefficients to a statistical level of significance. The large sample of students in the comparison group (students without autism) helps us still feel informed by these analyses. An a priori power analysis can use the effect size of the independent variables for the students without autism to calculate the estimated sample sizes required to detect a similar effect size in the autistic population. For the full sample size, a sample size of 233 was estimated for power of 0.95 and α of 0.05. Our sample of 170 corresponded with approximately 0.8 power. This means that, if the true effect of these motivation variables is the same for autistic students as it is for students without autism, then this analysis is estimated to have an 80% likelihood of successfully identifying that effect. However, this power shrinks considerably when considering individual variable contributions. Since the coefficients for each significant motivation relationship for students without autism were so small, the power to detect these values for the autistic group is also smaller.

The measurement of autism in this dataset utilized parent surveys asking if their child had ever had an autism diagnosis. This relies on parent knowledge of autism diagnoses which could potentially have changed in name over time for their child.

Missing data in this study were addressed through full information maximum likelihood analysis to derive all regression coefficients. This is a strong technique for handling missing data at least as good as multiple imputation (Allison, 2001).

Finally, it is important to understand how these variable were operationalized in the survey. As described in the variable section, survey questions ask specifically about the students’ math *classes*, not math in general. For example, the survey asks “What students learn in [fall 2009 math course] is useful for everyday life?” rather than “What students learn in math is useful for everyday life.” This is an important consideration when interpreting these variables. Since our outcome is final math GPA based on these courses, this operationalization was still appropriate for our model.

Conclusion and Future Research.

Overall, while our sample size of autistic students may not be large enough to capture small positive effects on math GPA of socioemotional variables seen in students without autism, we can be convinced that these models of achievement-related motivation behave differently for students with autism. Initial test scores do not seem to be nearly as important in positively predicting final math results, nor are subjective task value measures. Given the heterogeneity in students diagnosed with autism, it may also be important to consider grouping of individuals within the sample, which we have demonstrated in this analysis. Future studies could also aim to address some of the limitations of this study, such as using different operationalizations of variables and not relying on parent reports for autism.

We found that math identity may be a strong influencer of motivation for high school students with autism, but only for those with above average math scores. When intervening to improve outcomes for autistic students in high school math, it is likely important to intervene on factors influencing their beliefs in their own math ability and how much they perceive themselves as a math person for lower performing math students and higher performing math students respectively. Research interventions on these areas may shed more light on the causal effects of these socioemotional factors.

These new findings help improve our understanding of the forces affecting the math course-taking success of autistic students. Between the stereotypes involving math or quantitative skills in autism and the general differences between common autistic traits and those of their peers, it may be difficult to understand how these students are motivated in their high school math classes. Motivation frameworks such as expectancy value theory are traditionally employed to help us understand a wide variety of educational decisions and outcomes for non-disabled students – including decisions and outcomes regarding high school math course-taking and achievement (such as Andersen & Ward, 2014; Fong & Kramer, 2020). However, these models manifest differently for autistic students who appear to interpret and respond to their environments and experiences differently. Research is just beginning on the many aspects of academic motivation for autistic high school students. Investigating the effects of identity in different subjects, particularly for high-functioning or high performing but disabled students, is one avenue to explore to better understand their motivation.

## Electronic Supplementary Material

Below is the link to the electronic supplementary material.

Supplementary Material 1

## Footnotes

1 Please note that we generally aim to use the term “autistic” rather than “with autism” in this paper. This is in an effort to use identity-first language as it is currently the more preferred option by the autistic population (Kenny et al., 2016).

2 Note that this label is contested both for its generalization of autistic individuals and for its reliability as a distinct group (Alvares et al., 2020a, b).
